# Medical Students’ Experience in a Trauma Chaplain Shadowing Program: A Mixed Method Analysis

**DOI:** 10.1080/10872981.2019.1710896

**Published:** 2020-01-03

**Authors:** Sofia Gomez, Betty White, James Browning, Horace M. DeLisser

**Affiliations:** a Perelman School of Medicine, University of Pennsylvania, Philadelphia, PA, USA; b Department of Pastoral Care and Education, University of Pennsylvania Health System, Philadelphia, PA, USA

**Keywords:** Spirituality, Pastoral care, Chaplain shadowing, Religion, Interprofessional education, Physician-patient communication

## Abstract

Despite the importance of spirituality to health and patient care, there remains a lack of educational opportunities for medical students to learn about and engage the spiritual needs of patients. Shadowing of hospital chaplains has been employed as a means of providing instruction in spirituality, but published experiences of this pedagogy are limited. This study therefore analyzed an elective, first-year medical student, eight-hour, trauma chaplain shadowing experience, the objectives of which are to increase students’ knowledge and understanding of (i) the role of chaplains/pastoral care in patient care; (ii) strategies for engaging patients and/or families in difficult situations; and (iii) approaches for discussing issues of spirituality with patients and families. A questionnaire was sent to participants after the experience assessing the value of the experience. Two focus groups provided additional qualitative data. Of the 148 participants over 6 years, 100 completed the questionnaire (68%). Participants on average engaged 1.78 trauma patients or their families and experienced 3.63 overall patient/family interactions during their shadowing. Over 90% of respondents agreed or strongly agreed that the experience provided agreater understanding of the role of the chaplain, and was educationally, professionally, and personally useful. Over 60% of respondents agreed or strongly agreed that the experience improved their understanding of discussing difficult or spiritual topics with patients and families. Nearly all respondents (98%) would recommend asimilar shadowing experience to fellow medical students. Qualitative remarks echoed these findings, revealing themes surrounding the educational benefits, surprise, and awe experienced by participants, and indicating appreciation for the interprofessional aspect of the experience. These data demonstrate that trauma chaplain shadowing may be effective for introducing first-year medical students to healthcare chaplaincy, educating them about the challenges of navigating difficult spiritual conversations with patients and families, and exposing them to interprofessional collaboration.

## Introduction

Addressing the spiritual and religious needs of patients has been increasingly associated with positive clinical outcomes, decreased overall mortality rates, improved mental health, and increased quality of life [1]. In response to these data, major medical organizations including the Association of American Medical Colleges, the American Medical Association, the American College of Physicians, and the Joint Commission have all called on healthcare providers to address the spiritual needs of patients and their loved ones [2]. Further, the World Health Organization has included spirituality, alongside physical and psychosocial well-being, as a core dimension of palliative care [3]. In line with the efforts of these organizations, there has been the promulgation of national competencies to provide a framework for incorporating spirituality training into medical education [2].

Despite the increasing recognition of spirituality as a core facet of health and patient care, there continues to be a lack of educational opportunities for medical students to learn about and engage the spiritual needs of patients. A National Survey conducted in 2008 including responses from 122 US medical schools found that while 90% of schools offer training on spirituality and health, only 7% of schools require participation in a spirituality course [4]. This content, however, was typically taught in courses addressing different topics or in elective courses, workshops, and/or self-directed learning [5].

Healthcare chaplains play an important role in the clinical sphere, providing spiritual and pastoral care, presence, and counseling to patients, their families, staff, and others in the healthcare community [6,7]. Positive outcomes associated with chaplain care include relief of patient and family spiritual distress, improved communication between patients’ families and the medical team, reduced spiritual and emotional distress on the part of healthcare providers, and enhanced patient satisfaction with the care provided [8,9]. In fact, there are reports that chaplaincy services may lower mortality rates [10]. In 2015, 70% of hospitals surveyed provided pastoral care services, up from 53% in 2002, based on data from the American Hospital Association [11]. Given the nature of their work, observation of hospital chaplains provides a convenient, and potentially very informative, experience for medical students to learn about various aspects of patient spirituality. Despite its potential educational benefits, shadowing of hospital chaplains by medical students appears to be an underutilized pedagogy, with published reports providing very limited descriptions of the shadowing experience and/or reporting only on single cohorts of students [12–16]. In a previous report, we described our initial experience with a group of 21 first-year medical students who participated in the initial offering of a trauma chaplain shadowing experience [16]. In this earlier study, participants reported an increased understanding of how to engage patients and families in difficult conversations; learning about the chaplain’s role in the hospital; and finding the experience to be educationally valuable. In this current follow-up report, we describe outcomes from this trauma chaplain shadowing program that now encompasses nearly 150 students and 6 years of students’ experience.

## Methods

### Program description

During the fall semesters of 2013–2018, first-year medical students at the Perelman School of Medicine (PSOM) at the University of Pennsylvania were provided with the opportunity to participate in the Trauma Chaplain Shadowing Program, a collaborative educational initiative between the Perelman School of Medicine and the Department of Pastoral Care and Education Department of the University of Pennsylvania Health System. The shadowing is offered as an optional patient experience for the *‘Doctoring IA: Introduction to Medicine and Society,’* course, which explores the social, cultural, and structural influences on physician–patient interactions with an emphasis on personal introspection and relational communication [17]. The educational goals for the shadowing were to increase students’ knowledge and understanding of (i) the role of chaplains/pastoral care in patient care; (ii) how to engage patients and/or families in difficult situations; and (iii) how to appropriately discuss issues of spirituality with patients and families.

Students received a brief description of the shadowing program by a student coordinator at the start of a *‘Doctoring IA’* lecture, and subsequently received an e-mail with some brief general information about the role of a healthcare chaplain. This e-mail included a link whereby students were able to sign up for available shadowing shifts online on a first-come-first-serve basis. A participating student’s experience involved a 6- to 8-h shift, during an evening or on a weekend, shadowing an on-call trauma chaplain at the Hospital of the University of Pennsylvania (2013) or Penn Presbyterian Medical Center (2014–2018) in Philadelphia. The duties of these chaplains included responding to traumas in the emergency department, offering pastoral care services to patients and their family members, as well as providing chaplaincy services throughout the hospital. Throughout the shadowing, with the goals of the program in mind, the chaplain would process with the student the patient or family experiences they were encountering. A journal was available for students to anonymously write a reflection about their experiences immediately after the shadowing. Students were also invited to attend the chaplains’ daily debrief the subsequent morning to help process or discuss any of the emotions or challenging experiences they encountered during their shadowing.

### Program evaluation

After their shadowing experience, each participant was emailed a link to an electronic questionnaire, with two or three reminder emails sent to all students to encourage participation after the fall semester. The participants in the study were a convenience sample. We aimed for a response rate of 50%, on the upper end of what is often seen with physician surveys [18]. The questionnaire included 6 Likert-style questions that assessed the educational value of the program to the students. Survey and focus group discussion questions were created using language that would directly assess student satisfaction regarding the main educational objectives of the shadowing program, as outlined above (see ‘Program description’). Likert-style questions were designed to quantify participants’ subjective assessments of changes in their understanding of the chaplain’s role and ability to facilitate difficult conversations as well as perceived personal and educational usefulness of the program. The six questions were:

(i) ‘This experience provided me with a greater understanding of the role of chaplains in patient care'.
(ii) ‘This experience provided me with a greater understanding of how to engage patients and/or families in difficult conversations'.
(iii) ‘This experience provided me with a greater understanding of how to appropriately discuss issues of spirituality with patients and/or families'.
(iv) ‘This experience was useful for my medical education and/or career'.
(v) ‘This experience was useful for my personal development'.
(vi) ‘Debriefing with chaplains after each encounter was a valuable part of my shadowing experience'.

Students were also asked to indicate:

(i) The number of traumas observed, patient deaths experienced, and patients/families visited;
(ii) If there was time to debrief with a chaplain after any encounter (‘yes/no’);
(iii) If the participant would recommend this experience to other medical students (‘yes/no’);

Finally, students were asked to reflect on their shadowing experience and what they learned from it. The questions were based on the objectives of the educational experience, but not pilot-tested. Additionally, two discussion groups were held after the Fall of 2013 and 2014, during which a discussion about participants’ experiences took place with available participants and one male faculty physician moderator (H.D.), with 20 years of experience as a medical educator and in facilitating small group conversations. Respondents were asked to provide their age and gender, but no other identifying information was solicited.

Likert-style question results and other quantitative question results were analyzed using calculations of means, standard deviations (SD), and medians using Microsoft Excel. Percentages of male versus female gender and ‘yes/no’ questions were calculated. Two authors (S.G. and H.D.) qualitatively analyzed all free response question entries and full transcripts of the two focus groups, searching for common themes across the participants’ responses. The methodological orientation employed was a qualitative content analysis using inductive reasoning. There was no software assistance used for the data analysis. S.G. was a female medical student who had participated in the shadowing program in 2016 and coordinated student shadowing in the fall of 2017, interacting with participants via email for coordination of shadowing scheduling and survey completion. H.D. was present at both focus groups and was the course director of the Doctoring IA course for all first-year medical students included in the study. Qualitative analyses were exclusively performed using the de-identified focus group transcripts and free responses.

Focus groups were held in a classroom at the medical school, a familiar location for all students, in June 2014 (90 min) and February 2015 (60 min) for the Fall 2013 and 2014 participants, respectively. All shadowing participants from the Fall of 2013 and 2014 were invited to attend their respective focus groups. With the goal of facilitating an unstructured conversation, the moderator began the discussion by encouraging students to share ‘something memorable or impactful’ from their shadowing experience. Facilitation of discussion that followed was based on the topics raised by the students themselves. Each focus group was audio-recorded and subsequently transcribed by a commercial vendor. Transcripts of the conversations were not made available to participants for revisions at any time. Comments made by all students in attendance were included in the analysis.

This study was performed in accordance with the Institutional Review Board of the University of Pennsylvania, and was deemed to be exempt given minimal risk to participants. Ethical considerations included voluntary participation of participants and all respondents were informed in written form (via e-mail) that their survey responses would be reported in aggregate and no individual would be identified.

## Results

### Student demographics

Of the 148 students who participated in the trauma chaplain shadowing program over the 6 years of data collection, 100 students completed the electronic questionnaire (68%). Both the participation number and response rates varied over the years (Table 1) with the last two years (Fall of 2017 and 2018) containing the highest participation numbers and response rates. Of respondents, 63% were female, 37% were male. The average age was 24 years old, with an SD of 2 years.

**Table 1.** Trauma chaplain shadowing participation and response rates from 2013 to 2018

Year	Number of respondents	Number of students who participated	Response rate (%)
2013	19	39	49
2014	17	27	63
2015	10	21	48
2016	10	15	67
2017	23	24	96
2018	21	22	95

### Student experiences

The number of traumas encountered during the shift ranged from 0 to 7, with a mean of 1.78, SD of 1.6, and median of 1. The number of overall patient or family interactions encountered during the shift ranged from 0 to 12, with a mean of 3.63, SD of 2.6, and a median of 3. While 20% of respondents reported encountering no trauma patients or families of trauma victims during their shift, 98% of students encountered at least one hospitalized patient or family with their chaplain. During their shadowing, 18% of respondents reported observing a death. Importantly, 95% of respondents noted having an opportunity to debrief their experiences with their assigned chaplain during their shift. Additionally, 98% of respondents would recommend a similar trauma chaplain shadowing experience to a fellow medical student at some point during their medical education.

### Student assessments of the educational value of the shadowing experience

The Likert-style questions employed a 5-point scale (1 = strongly disagree, 5 = strongly agree), and included statements regarding the usefulness and value of the shadowing experience to the respondents (Table 2). Respondents scored the statement ‘this experience provided me with a greater understanding of the role of chaplains in patient care’ at a 4.72 (SD 0.62), with a median of 5; ‘this experience provided me with a greater understanding of how to engage patients and/or families in difficult conversations’ at a 4.11 (SD 1.03), with a median of 4; ‘this experience provided me with a greater understanding of how to appropriately discuss issues of spirituality with patients and/or families’ at a 3.78 (SD 1.15), with a median of 4; ‘this experience was useful for my medical education and/or career’ at a 4.58 (SD 0.71), with a median of 5; ‘this experience was useful for my personal development’ at a 4.54 (SD 0.66), with a median of 5; ‘debriefing with chaplains after each encounter was a valuable part of my shadowing experience’ at a 4.41 (SD 0.85), with a median of 5.

**Table 2.** Summary of participant responses to Likert-style (1 = strongly disagree, 5 = strongly agree) questions on respondents’ trauma shadowing experience

Item	Mean (SD)	Median
‘This experience provided me with a greater understanding of the role of chaplains in patient care.’	4.72 (0.62)	5
‘This experience provided me with a greater understanding of how to engage patients and/or families in difficult conversations.’	4.11 (1.03)	4
‘This experience provided me with a greater understanding of how to appropriately discuss issues of spirituality with patients and/or families.’	3.78 (1.15)	4
‘This experience was useful for my medical education and/or career.’	4.58 (0.71)	5
‘This experience was useful for my personal development.’	4.54 (0.66)	5
‘Debriefing with chaplains after each encounter was a valuable part of my shadowing experience.’	4.41 (0.85)	5

### Students’ qualitative comments on the shadowing from the post-shadowing survey

There were 34 entries (out of 100 respondents) to the free response question recorded. Five themes arose from these responses. The first was a sense of awe regarding their participation: respondents used words such as ‘amazing', ‘impactful', ‘fantastic', ‘wonderful', and ‘excellent’ to describe their experience. Second, students noted observing ‘sensitive’ patient and family situations; one described witnessing ‘a terrible and vulnerable moment.’ A third theme referenced the learning students experienced during the shadowing. ‘[I learned] a lot watching’ the chaplain ‘navigate the role of trauma chaplain and engage families and patients.’ Another student reflected on the benefits of interdisciplinary and interprofessional collaboration embodied in her shadowing noting: ‘Being able to take off my clinical hat and put on my pastoral care hat allowed me to really experience what it’s like to empathize and connect with patients and their families.’ A fourth theme involved appreciation. Multiple respondents explicitly noted a sense of ‘appreciation’ for being granted the opportunity to participate in the program and to observe pastoral care at work in such vulnerable patient experiences. Other respondents expressed gratitude for the personal time they were able to spend with their assigned chaplain to better understand their backgrounds and perspectives. One student noted, ‘I … [had the] the chance to have a long and wonderful conversation with the chaplain on call,’ while another said, ‘Chaplain [deidentified] is amazing and really helped me process my own feelings.’ Finally, in reference to variability in the timing and intensity of clinical encounters, several respondents expressed a desire to ‘see and experience more.’ One student noted, ‘The night that I shadowed, there was not a single trauma case.’ Another reported that their shift was ‘pretty slow’ and they would have benefitted from shadowing ‘at busier times.’

### Student focus group reflections on the shadowing experience

Two focus groups (six student participants at Fall 2013 group, 12 student participants at Fall 2014 group) were held to further explore student experiences shadowing the trauma chaplains. Four major themes were identified. First, students unanimously mentioned the value of watching chaplains manage difficult patient or family situations. One student noted, ‘I would echo the sentiments of [others] in that [the shadowing experience] was instructional on how to be present during suffering.’ Multiple examples of chaplains attending to patients’ emotional or spiritual needs better than other providers were described:

Honestly, the chaplain did a better job than I think [the physicians] could have.

The chaplains are very good at certain things that the doctors don’t have the time for or are just not good at.

I definitely feel like the chaplain … provided me with a lot of things that I was missing … It was like I was learning to be a doctor.

Second, the educational benefits regarding the role of the chaplain were also discussed. A student commented: ‘The other thing that really stuck with me is how much one can learn from the chaplain side of things.’ Another student noted that the shadowing experience brought to light ‘the interdisciplinary nature of being a chaplain.’ Further, participants noted it was helpful to be in the chaplain’s ‘shoes,’ or ‘role,’ and expressed a desire for similar programs extending to other fields such as nursing. A third theme was a shared sense of surprise about the variety of important roles played by healthcare chaplains, including providing pan-religious (including non-Judeo-Christian religions, specifically) and spiritual support, contacting family members, identifying trauma victims, funeral planning, emergency room security, note-writing, and acquiring health records. In this regard, students commented:

Going into the experience with my perceptions on what a chaplain did versus what they actually do, it’s very different. They’re actually a lot more multi-faceted … So that’s one thing that really stuck with me.

I didn’t expect the role of a chaplain to be one of connecting the medical team to the patient and their family.

One of the things … I don’t think I would have really thought of is that chaplains could be good [support] to the staff.

My prior conception was that the chaplain primarily visited patients for religious needs, [but] almost every call that the chaplain [had] was non-religious … She prayed with a couple of patients, but otherwise, it was trying to contact families or mediating conversations with families.

Finally, students commented on how the chaplains were welcoming and accepting of them: One student mentioned, ‘[The chaplain] was incredibly accommodating and I really felt like, she took me under her wing;’ while another said, ‘I felt like it was like us two, like we were hanging out together, you know, two peas in a pod.’ A student who attended the chaplains’, post-call, morning debrief stated, ‘It felt really nice to be appreciated, because all the chaplains were like, “Thank you so much for coming and shadowing!” As a med[ical] student, you don’t feel appreciated very much'.

## Discussion

Recent literature has provided evidence of the importance of addressing patients’ spiritual and emotional needs as a key component to their medical care to improve health outcomes and quality of life [1,3]. Although initiatives to encourage spirituality as a core facet of medical education have begun [2], their implementation has been limited [4], and the effects of these initiatives have been poorly investigated due to small sample sizes of participants [5,12–16]. To the best of our knowledge, this paper is the first to describe the educational impact of a trauma chaplain shadowing program for medical students using a mixed-methods approach based on a large number of students (100) and over several (six) years of student cohorts. This study presents robust data indicating clear benefits of a trauma chaplain shadowing program with respect to medical student education on engaging in difficult patient conversations involving spirituality and on the importance of interdisciplinary care.

Among the respondents, the shadowing was seen as an experience that benefitted them educationally, professionally, and personally; they valued the clinical exposure and the interactions with chaplains and patients/families. This finding is in line with our pilot study of the first year of the shadowing program [16]. More importantly, however, our results indicate that this shadowing program met each of the three delineated aims.

First, participants gained knowledge of healthcare chaplaincy services and pastoral care, evidenced by quantitative data indicating a greater understanding of the chaplain’s role as well as the qualitative data that indicated common themes of learned knowledge and surprise surrounding the chaplain’s diverse set of roles in the hospital setting. These findings are significant given studies documenting physicians’ incomplete knowledge of the training, scope of practice, and skills of professional chaplains [8,19,20], even among palliative care providers [21]. Similarly, published findings show that in many intensive care units, chaplains are an underutilized resource [22]. Our data, therefore, highlight the potential value of a robust chaplain shadowing program in increasing medical trainees’ understanding and appreciation of the value of chaplaincy to clinical care.

Second, students reported increased knowledge and understanding of how to engage patients/families in difficult situations. Specifically, students came to appreciate that the chaplains have a distinct set of communication and relational skills that physicians often lack, and which they could, and should, incorporate into their future careers as practicing physicians. With the recognition of the need for additional approaches to promote medical trainee empathy and relational competence [23], chaplain shadowing provides a unique teaching opportunity to model and reinforce essential physician interpersonal skills.

Finally, our trauma chaplain shadowing program enabled students to gain a greater understanding of how to engage the spirituality of patients and/or families. This is certainly a relevant finding given the overall inadequate training medical students receive in addressing patient spirituality [4,5]. As medical educators seek to address these deficiencies, our data, as well as that of others [24], support the inclusion of a structured shadowing program as an important element of curricula for engaging the spirituality of patients.

The program also showed evidence of additional effects beyond achieving core objectives. The value of interprofessional collaboration with chaplains, as well as the benefit of observing the chaplains provide interdisciplinary care to patients, was mentioned by participants. Although interprofessional care and communication have been shown to be critical to effective patient care [25,26], there are still barriers to achieving this in the healthcare setting [27]. Further, medical education on addressing these barriers and improving interprofessional communication is still inadequate [28]. These data suggest that chaplain shadowing may also be an effective means to introduce medical students to the benefits of effective interprofessional collaboration. These promising findings have prompted investigation into the participating chaplains’ experiences in the program, including efforts to obtain their assessments of the shadowing’s impact on the medical students as well as on the chaplains themselves.

Several limitations of this study are noted. All participants were first-year medical students from a single institution; thus, the applicability of our findings to students at other medical schools or upper-level medical students still needs to be confirmed. Additionally, because the program was elective, there may be an element of selection bias for students with an interest in spirituality, who might be more likely to view this experience positively. More distal follow-up data would be useful for putting the value of the experience into perspective in terms of overall curricular benefit. Finally, the qualitative data were elicited from only a subset of all the participants; caution should be exercised in generalizing these findings.

Furthermore, as noted by many participants, the short time available for shadowing, the different levels of experience among chaplains, and the nature of the program involving unpredictable patient needs meant that the experience was non-standardized. For example, there was a wide spectrum in the number of patient or family interactions encountered by the students, ranging from 0 to 12. Variation in student experiences makes it difficult to generalize the data collected. Future directions therefore at PSOM include piloting a spring semester shadowing program and broadening the shadowing experience to incorporate multiple shifts in anticipation of establishing the shadowing experience as a requirement for all students. In this regard, we note that, at PSOM, a certificate program in spirituality and health has already been established for students seeking more exposure and instruction in this area.

Despite these limitations, this study does provide evidence that a trauma chaplain shadowing program for first﻿-year medical students may be an effective means of introducing students to healthcare chaplaincy services, educating them on the challenges of navigating difficult spiritual conversations with patients and families, and exposing them to interprofessional collaboration. Further work should include a broader range of medical students across pre-clinical and clinical years, as well as students who may not have a pre-existing interest in spirituality, to further investigate the effectiveness of this type of program in medical education.
